# Integrated molecular and metatranscriptomic evidence of Tacaribe virus and the brain virome profile of *Molossus molossus* bat sampled in Brazil

**DOI:** 10.1128/spectrum.01960-25

**Published:** 2026-01-21

**Authors:** Larissa Leão F. de Sousa, Rodrigo Lopes Sanz Duro, Raquel de Oliveira Vaz, Clara Lacerda de Athayde, Junior Olimpio Martins, Bruna Stefanie Silvério de Lima Slobosk, Mariana Dias Guilardi, Gustavo Cabral-Miranda, Edmilson Ferreira de Oliveira-Filho, Jan Felix Drexler, Luiz Mário Ramos Janini, Ricardo Durães-Carvalho

**Affiliations:** 1 Department of Microbiology, Immunology and Parasitology, Federal University of São Paulo 28105, São Paulo, Brazil; 2 Rabies Diagnosis Laboratory, Central Laboratory of Public Health - LACEN, Fortaleza-CE, Brazil; 3 Department of Morphology and Genetics, Federal University of São Paulo 28105, São Paulo, Brazil; 4 Interunit Bioinformatics Graduate Program, Institute of Chemistry, University of São Paulo 153989, São Paulo, Brazil; 5 Anhembi Morumbi University 67917, São Paulo, Brazil; 6 Institute of Biomedical Sciences, University of São Paulo, São Paulo, Brazil; 7 Charité-Universitätsmedizin Berlin, corporate member of Freie Universität Berlin and Humboldt Universität zu Berlin, Institute of Virology, Berlin, Germany; Barnard College, Columbia University, New York, New York, USA

**Keywords:** Tacaribe virus, *Molossus molossus*, metatranscriptomic, Brazil

## Abstract

Tacaribe virus (TCRV), a New World arenavirus, is associated with neotropical frugivorous bats, particularly *Artibeus* spp., and is considered to have zoonotic potential. Here, we report the detection of TCRV in multiple biological compartments of a *Molossus molossus* (velvety free-tailed bat), an insectivorous species commonly found in Brazilian urban ecosystems. Brain tissues negative for rabies were subjected to high-throughput RNA sequencing, revealing a diverse array of viral taxa, including partial L and S segments of TCRV with respective genome coverages of 68.2% and 65.6%. These sequences shared 90% nucleotide identity and 94% amino acid identity with TCRV reference strains. Phylogenetic reconstruction grouped the newly identified TCRV sequences and the TCRV strain A354 (isolated from a Brazilian *Artibeus planirostris* bat) within a clade that also includes Tietê mammarenavirus strains (Brazilian *Carollia perspicillata* bat), both of which are genetically divergent members of the Arenaviridae family. The detection of TCRV in *M. molossus* may indicate previously unrecognized circulation of this virus in insectivorous bats, expanding our understanding of its tissue tropism and host range. This finding is particularly significant given the synanthropic behavior of *M. molossus* bats and its potential implications for TCRV evolution. Moreover, our results highlight the critical role of untargeted high-throughput sequencing in uncovering overlooked viral diversity, enabling the detection of unexpected pathogens in non-traditional hosts and tissues. While TCRV remains poorly characterized in terms of human pathogenicity, continued surveillance is warranted to assess potential spillover risks.

**IMPORTANCE:**

Understanding which animals carry viruses is key to predicting and preventing disease spread. Here, we report the first detection of Tacaribe virus (TCRV), previously found only in fruit-eating bats, in multiple biological compartments, including the brain, of *Molossus molossus*, an insect-eating bat common in urban areas of Brazil. This finding expands the known range of both the virus and its possible hosts. By confirming this unexpected host-virus association using original tissue samples, our study provides new insights into how TCRV may circulate in nature. While TCRV is not currently considered a major threat to humans, its detection in a new bat species raises important questions about its transmission, evolution, and potential health impacts. These results emphasize the importance of monitoring diverse bat species, including those not traditionally linked to specific viruses, to better assess emerging virus risks in changing environments.

## OBSERVATION

Tacaribe virus (TCRV) is a New World arenavirus (NWA) not known to naturally infect humans; however, rare cases of laboratory-acquired infection associated with mild, flu-like symptoms have been reported, suggesting that TCRV may have zoonotic potential (1). NWAs are divided into Clades A, B, C, and A/Rec (D), with all known human pathogenic NWAs, such as Junín (JUNV), Machupo (MACV), Guanarito (GTOV), Chapare (CHAPV), and Sabiá (SABV) viruses, belonging to Clade B and restricted to South America (2, 3). Unlike most NWAs, which are rodent-associated, TCRV, classified within Clade B, was first isolated between 1956 and 1958 from *Artibeus* fruit bats and mosquito pools in Trinidad and Tobago (4, 5). More recently, divergent mammarenaviruses have also been identified in distinct hosts, such as pikas (*Ochotona* genus) and hedgehogs (*Erinaceus* genus), further expanding their known host range (6–8).

In the Dominican Republic, full-genome sequences of TCRV were successfully obtained from an *A. jamaicensis* bat collected in 2014, with viral RNA detected in multiple tissues (9). These sequences showed 83.3%–86% nucleotide and 91.8%–93.7% amino acid identity compared with the TCRV strain TRVL-11573 sampled from a frugivorous bat. In Brazil, surveillance efforts targeting neotropical bats revealed TCRV sequences in *Artibeus* spp. and a novel arenavirus, Tietê mammarenavirus (TEVT) in *Carollia perspicillata*, suggesting that New World frugivorous bats may harbor a broader diversity of arenaviruses within the TCRV serogroup (10). Additionally, this study provided evidence that arenavirus infection in bats can be systemic, as evidenced by the presence of the virus in the intestine, followed by the spleen, lungs, liver, and kidneys.

Here, we analyzed 20 brain samples collected between 2022 and 2023 in the state of Ceará, northeastern Brazil, through passive surveillance activities conducted under the Rabies Surveillance and Control Program coordinated by the Central Public Health Laboratory of Ceará (LACEN/CE). All samples were sent to a BSL-3 facility, treated with lysis buffer, and held for 24 h before downstream processing.

Each sample was processed individually and subsequently grouped into four pools of five individuals each. The pooling strategy was performed according to the following criteria: samples were combined only when they belonged to the same bat species and had tested negative for rabies virus (RABV), as evidenced by direct immunofluorescence assay (DFA), mouse inoculation test (MIT), and PCR (11). Thus, pools were species-specific (i.e., *Desmodus rotundus* [common vampire bat] and *M. molossus* were not mixed), preserving host-species resolution at the pool level. For metatranscriptomic profiling, the rabies-negative samples underwent library preparation with the Zymo-Seq RiboFree Total RNA Library Prep Kit, following the manufacturer’s protocol. Library construction was confirmed using Agilent’s D1000 ScreenTape Assay on a TapeStation and sequenced with the Illumina NovaSeq 6000 platform at a depth of 40 million paired-end reads (150 bp).

**Fig 1:**
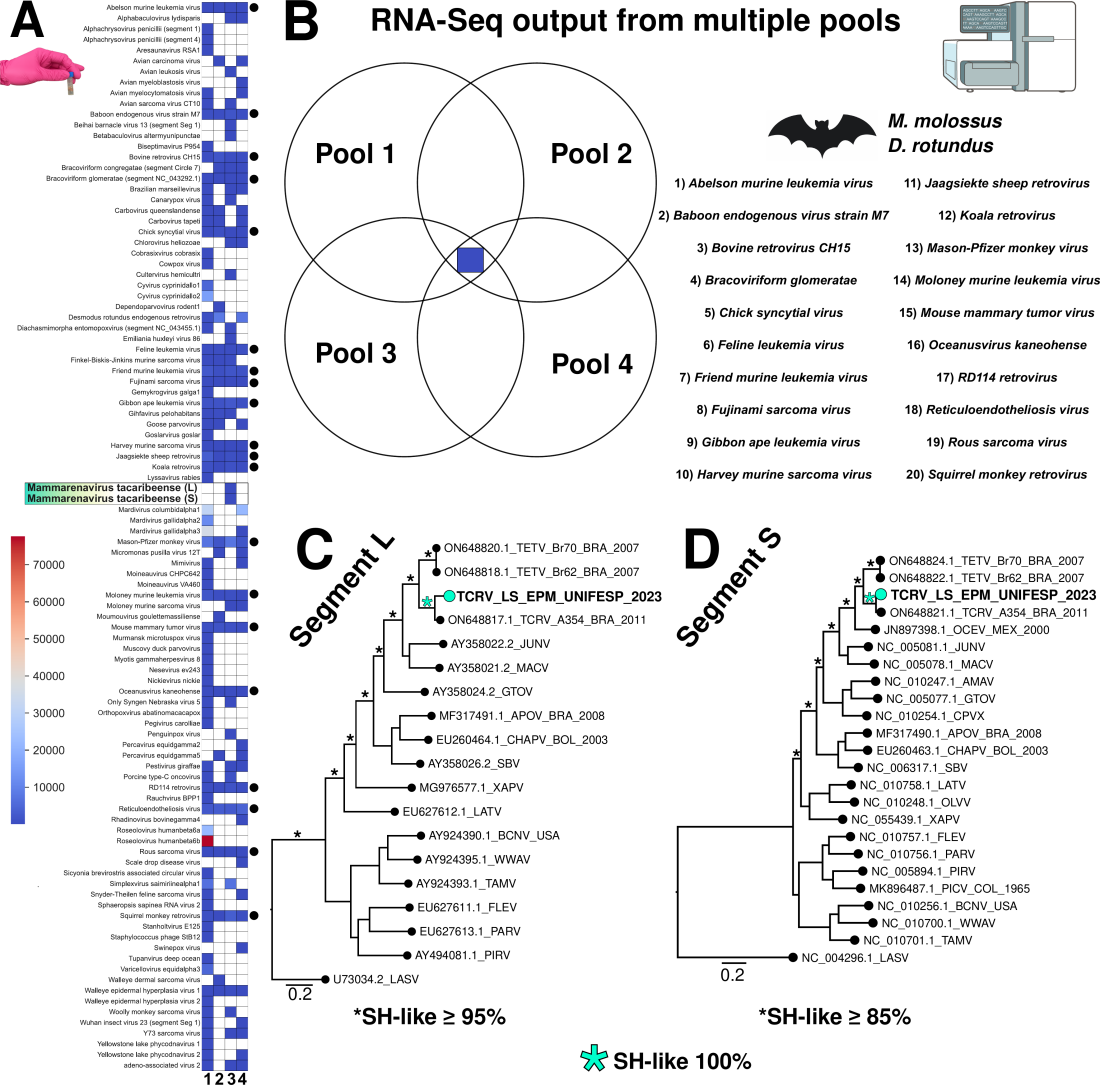
Viral metatranscriptomic profiles obtained from multiple brain pools and evolutionary analysis of TCRV identified in our study. The heatmap shows viral detection profiles across four distinct pools, based on assignment scores from RNA-Seq reads. Each row represents a distinct identified virus, and columns 1 to 4 correspond to the different analyzed pools. Color intensity indicates the absolute number of mapped reads for each virus in each pool. Black dots on the right side indicate viruses detected in all four pools analyzed (**A**). Venn diagram showing the distribution and overlap of viral species identified across four pools derived from *Molossus molossus* (pools 1 and 3) and *Desmodus rotundus* (pools 2 and 4), respectively. The blue square in the central region represents twenty viral species shared among all four pools, which are listed on the right side (**B**). Maximum-likelihood phylogenetic trees based on L (3,454 nt) (**C**) and S (1,787 nt) (**D**) segment reads of *Mammarenavirus tacaribeense* obtained from RNA-Seq. Sequences identified in this study are highlighted in cyan. Branch support values are based on the Shimodaira-Hasegawa (SH-like) test. Branch lengths are scaled to nucleotide substitutions per site. Tacaribe virus (TCRV), Tietê mammarenavirus (TETV), Ocozocoautla virus (OCEV), Junín virus (JUNV), Machuco virus (MACV), Amaparí virus (AMAV), Guanarito virus (GTOV), Cupixi virus (CPVX), Aporé virus (APOV), Chapare virus (CHAPV), Sabiá virus (SABV), Latino virus (LATV), Oliveros virus (OLVV), Xapuri virus (XAPV), Flexal virus (FLEV), Paraná virus (PARV), Pirital virus (PIRV), Pichindé virus (PICV), Bear Canyon virus (BCNV), Whitewater Arroyo virus (WWAV), Tamiami virus (TAMV), Lassa virus (LASV). Countries: BRA, Brazil; MEX, Mexico; BOL, Bolivia; COL, Colombia; USA, United States of America.

Our analysis revealed a diverse viral landscape in brain tissue from *M. molossus* and *D. rotundus*, two bat species known to carry zoonotic and potentially pathogenic viruses (12, 13). The viral diversity, depicted through multiple visualization methods, heatmaps, Venn diagrams, and maximum-likelihood phylogenetic trees, offers a comprehensive view of viral presence and evolutionary relationships (Fig. 1). Also, based on RNA-Seq read counts and BLAST results, the heatmap highlights the presence of numerous viral taxa of medical and veterinary relevance, including arenaviruses, lyssavirus, and retroviruses (Fig. 1A). Notably, these included known endogenous retroviruses, such as koala retrovirus, Jaagsiekte sheep retrovirus, and mouse mammary tumor virus. Their presence may reflect either ancient viral integrations or ongoing low-level expression of endogenous elements, phenomena well-documented in vertebrate genomes, particularly in bats (14–16). Importantly, the consistent presence of a core group of 20 viruses across all pools (black dots and Venn diagram in Fig. 1B) might raise the hypothesis of a stable virome component in bat brain tissues, which likely represents common components of the chiropteran virome (17, 18).

**Fig 2:**
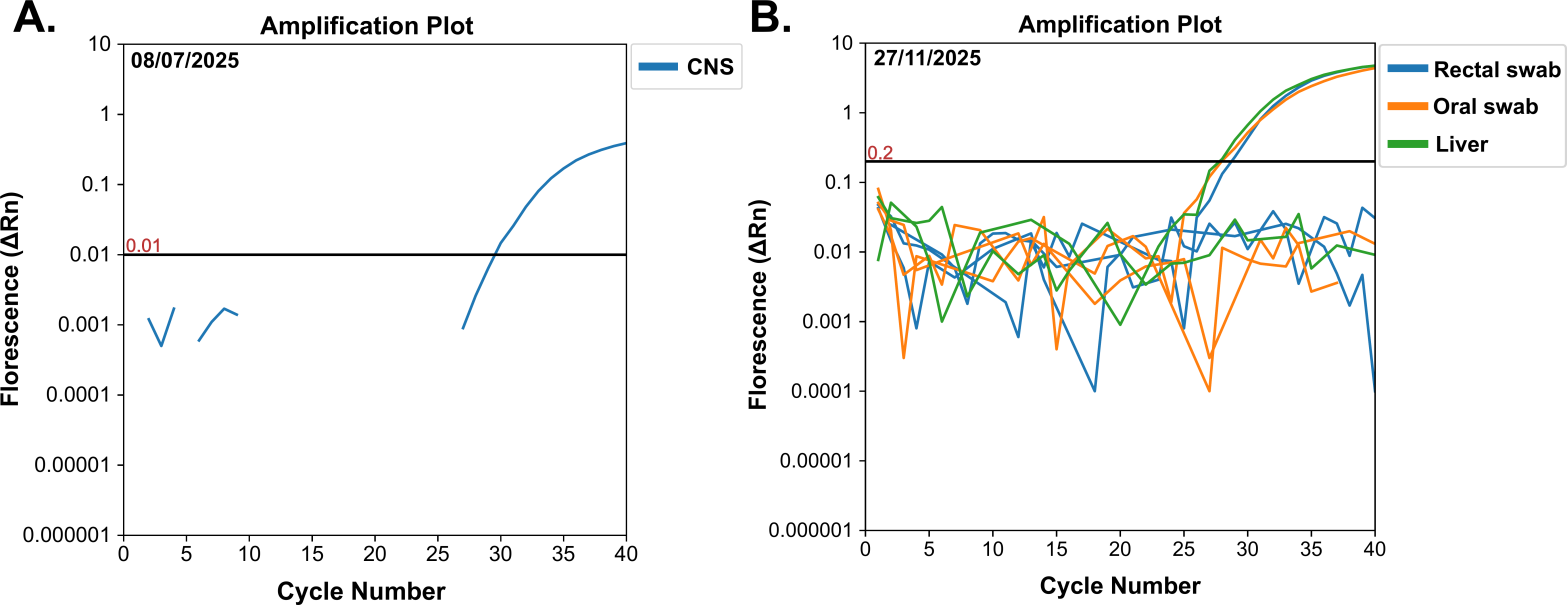
RT-qPCR amplification plots for TCRV detected in *M. molossus* (velvety free-tailed bat). Panel (**A**) shows the result of screening obtained from brain tissue (CNS), while panel (**B**) shows the results from rectal and oral swabs, and liver tissue. Each color represents TCRV detection in a distinct sample type, as indicated in the figure legend. Black horizontal lines represent the threshold point.

Additionally, we recovered *Mammarenavirus tacaribeense* (TCRV) from a *M. molossus* bat. Using the Genome Detective tool (19), we mapped 104 reads, achieving a coverage length of 4,844 nt (11 contigs) for the TCRV L segment, with 68.2% genome coverage and a coverage depth of 2.9×. This segment showed 90.2% nucleotide identity and 94.7% amino acid identity to the TCRV reference segment (NC_004292.1). On the other hand, the S segment yielded 40 reads, with a coverage length of 2,252 nt (6 contigs), 65.6% genome coverage, and a coverage depth of 2.4×. It also showed 90.1% nucleotide and 93.9% amino acid identity with the reference genome (NC_004293.1). Although new primers were designed and molecularly validated to extend the recovered segments to full length, we were not able to obtain them. We also recovered frozen rectal (Ct 28.7) and oral (Ct 28.0) swabs, along with a liver sample (Ct 27.7), all of which tested positive for TCRV by real-time qPCR (9). The brain tissue sample showed a Ct value of 29.5 (Fig. 2).

TCRV is an NWA previously isolated from *Artibeus* bats, but its detection in *M. molossus* raises intriguing questions about host range and potential spillover dynamics (20). Furthermore, the highly supported maximum-likelihood trees based on L and S segments (Fig. 1C and D) show that the TCRV sequences from this study cluster closely with the divergent arenavirus strain A354 from Brazil, sampled in 2011 from *A. planirostris*. This finding provides further evidence for classifying our sequences as TCRV and indicates evolutionary continuity or shared recent common ancestry among genetically distinct strains circulating in distinct bat hosts (21). This evolutionary pattern supports the hypothesis of localized diversification of TCRV in Brazilian bats and emphasizes the importance of bats as reservoirs for arenaviruses warranting further exploration (2). Species-focused surveillance will be necessary to clarify the role of *M. molossus* in TCRV ecology and to determine whether our finding reflects a single incidental detection or a sustained host-virus association.

The identification of TCRV in multiple biological compartments, including the brain of *M. molossus*, raises novel questions about the pathogenesis and host range of this virus, suggesting tissue tropism and potential for neurological involvement in natural hosts, a feature observed in zoonotic arenaviruses, such as JUNV and Lassa (22). These findings underscore the importance of expanding arenavirus surveillance beyond frugivorous bat species to include insectivorous taxa, which are abundant in human-modified landscapes and may contribute to arenavirus maintenance and transmission. Moreover, although TCRV is not currently linked to human disease, the phylogenetic proximity to pathogenic arenaviruses and demonstrated ability to replicate in mammalian cells highlights the importance of ongoing surveillance (10, 23).

To our knowledge, this study presents the first detection of TCRV in *M. molossus*, an insectivorous and synanthropic bat species highly adapted to urban environments, where it thrives due to stable food sources and shelter availability (24). This finding is particularly concerning, as bats are increasingly recognized as potential reservoirs of arenaviruses (10). The detection of TCRV in multiple tissues of distinct bat species living in close proximity to humans highlights a continued risk of viral spread (1, 2, 4). Although the specific barriers preventing zoonotic transmission remain undefined, these results underscore the urgent need to strengthen arenavirus diagnosis and surveillance efforts in bats throughout Brazil and Latin America.

The limitations of this study, despite employing high-throughput metatranscriptomic sequencing and newly designed primers, lie in the limited genomic coverage and sequencing depth of the recovered TCRV segments. Nevertheless, our approach successfully enabled the identification of the virus and expanded the known host range of TCRV beyond frugivorous bats, contributing to the limited genomic data for bat-derived TCRV. Importantly, we obtained a phylogenetically informative analysis through maximum-likelihood reconstruction, which yielded a well-supported and resolved tree topology. This placed the newly detected TCRV sequences alongside TCRV strain A354 and within a clade shared with TETV strains, providing strong evidence for the circulation of genetically divergent TCRV variants in northeastern Brazil.

Finally, our study extends beyond the analysis of sequencing libraries by situating the findings within an active state-level bat surveillance system that routinely collects brain tissue for rabies diagnostics. By integrating surveillance-derived sampling with metatranscriptomic screening, targeted molecular assays, and phylogenetic contextualization, this study provides valuable insights into TCRV host range and tissue involvement. Together, these elements demonstrate that the work combines surveillance, molecular confirmation, and evolutionary analysis, yielding a novel host-virus-tissue association and offering meaningful contributions to arenavirus ecology and future surveillance strategies.

## Data Availability

Complete raw sequencing data can be accessed through the repository https://zenodo.org/records/17787815. Additional data underlying the results presented in in the study can be accessed through the repository https://github.com/rduraescarvalho/TCRV. The sequences have been deposited in the NCBI BioSample database (http://www.ncbi.nlm.nih.gov/biosample/) under the UNIFESP Viral Epidemiological Surveillance project, with the following accession numbers: SAMN48756867 (TCRV L segment: 5,355 nt [75.4%]) and SAMN48756868 (TCRV S segment: 2,561 nt [74.6%]).
